# Milk Production, Milk Quality, and Behaviour of Dairy Cows Grazing on Swards with Low and High Water-Soluble Carbohydrates Content in Autumn: A Pilot Trial

**DOI:** 10.3390/ani9121012

**Published:** 2019-11-21

**Authors:** Verónica M. Merino, Oscar A. Balocchi, M. Jordana Rivero

**Affiliations:** 1 Department of Animal Sciences, Faculty of Agronomy, Universidad de Concepción, PO Box 160-C, Concepción 4030000, Chile; veronicamerino@udec.cl; 2 Institute of Animal Production, Faculty of Agricultural Sciences, Universidad Austral de Chile, PO Box 567, Valdivia 5090000, Chile; obalocch@uach.cl; 3 Departamento de Ciencias Agropecuarias y Acuícolas, Facultad de Recursos Naturales, Universidad Católica de Temuco, 4780000 Temuco, Chile; 4 Rothamsted Research, North Wyke, Okehampton, Devon EX20 2SB, UK

**Keywords:** high-sugar grass, perennial ryegrass, grazing behaviour, milk urea, nitrogen use efficiency, pasture management

## Abstract

**Simple Summary:**

Improving the performance of grazing systems and reducing their environmental impact are crucial in the current and future world contexts. In temperate grazing dairy systems, perennial ryegrass is widely used. Its nutritional composition can by modified via cultivar selection and management. We tested two swards: (1) a high-sugar cultivar submitted to fertilisation and defoliation regimes aimed at increasing sugar content and sugar-to-protein ratio and (2) a standard cultivar submitted to fertilisation and defoliation regimes aimed at decreasing sugar content and sugar-to-protein ratio. We tested these two swards with mid-lactation dairy cows grazing in daily strips for nine days in autumn and measured the amount of forage offered and consumed, the milk production and composition, and the grazing behaviour between morning and afternoon milkings. We found that the amount of residual herbage was greater in the standard cultivar sward, but the herbage consumed per cow was similar. Cows spent less time grazing in the high-sugar sward, but no difference was observed in the rumination time. Milk production and composition were similar between the two groups. This could imply that good-quality pastures would require a greater difference in nutritional composition to have an impact on animal performance.

**Abstract:**

Grazing ruminant systems can be sustainably intensified by improving efficiency while reducing their environmental impact. The objective of the present study was to examine the potential of pastures differing in water-soluble carbohydrates (WSC) and crude protein (CP) contents to affect milk production and composition as well as the behaviour of cows grazing perennial ryegrass (PRG) swards. By modifying the nitrogen (N) fertilisation rate (83 and 250 kg/ha per year) and the defoliation frequency (two or three leaves per tiller) in combination with cultivar selection (high-sugar vs. standard cultivars), we obtained two swards differing in WSC and CP contents. The two contrasting swards were each grazed by six dairy cows in nine daily strips in autumn. Pasture samples were collected to determine herbage mass and quality. Cow behaviour was recorded by direct observation. Herbage offered and apparently consumed were similar between swards (averaging 37.3 and 18.2 kg/cow, respectively), although the residual was lower in the high-sugar sward (1735 vs. 2143 kg/ha). Cows spent less time grazing in the high-sugar sward (66.9% v. 71.6%), but the rumination times was similar (14.6%). Milk production and composition were similar between groups, suggesting that high-quality pastures would require a greater difference in nutritional composition to affect animal performance.

## 1. Introduction

Grazing-based dairy production systems are commonly found in many temperate regions, such as southern Chile, where perennial ryegrass (*Lolium perenne* L.) is the most commonly used forage species [1]. It is well known that CP supply usually exceeds the animal requirements in cows fed on high-quality pastures, whereas the energy is the main limiting nutrient [2]. This imbalance between the supply of energy and protein can lead to an inability of the rumen microbial populations to capture the nonprotein N, which increases the dietary N excreted via urine as ammonia and reduces N use efficiency. Moreover, when dairy cows are fed protein in excess of requirements, the energetic cost associated with excreting N through urea synthesis increases, representing an energy loss that could have potentially been available for milk production [3]. 

Increasing WSC content could optimise rumen microbial protein synthesis, improving the efficiency of ruminal ammonia and dietary N utilisation for milk production [4,5,6], with the additional potential environmental benefit of reducing nitrous oxide emissions and nitrate leaching [7]. Moreover, more WSC content in grass could increase the proportion of glucogenic volatile fatty acids (i.e., propionate) in the rumen, thus increasing milk protein concentration [8]. Greater WSC content of PRG can be achieved by agronomic management, i.e., by decreasing the frequency of defoliation based on the leaf regrowth stage or decreasing the N fertilisation rate [9,10], or through genetic selection for greater levels of sugars in grass [11]. Nevertheless, the expression of high-sugar traits cannot be assumed to occur under all conditions, reflecting the influence of a genotype–environment interaction [11,12,13]. 

Several studies have focused on identifying the effect of high-sugar grass (HSG) on dry matter intake (DMI) and milk production of dairy cows, and most of them were performed under stall-feeding conditions. Moorby et al. [14] reported that increased digestible DMI of early-lactation dairy cows were achieved from HSG without effects on milk yield. In contrast, Miller et al. [6] reported a greater milk production from late-lactation dairy cows when the WSC content of the diet was increased through the use of HSG forage without significant differences in DMI. In both studies, the differences in WSC content between treatments were manipulated by either harvesting the forage considering the “time of day” variation [14] or applying contrasting N fertilisation rates [6]. In another indoor feeding study, neither the grazing behaviour and DMI nor the milk production and composition of mid-lactation dairy cows were improved by an increased WSC content in grass [15]. 

The effect of WSC and CP contents of forage on the behaviour of dairy cows in grazing conditions was studied recently by Rivero et al. [16]. The authors found that cows preferred to consume and spend more time grazing on swards with greater contents of CP instead of greater WSC. This was achieved with greater fertilisation rates (equivalent to 250 kg N/ha per year) and more frequent defoliations (at the stage of two leaves per tiller), indicating a preference for swards with certain nutrient value. Understanding how the nutritional value of HSG influences grazing behaviour and hence herbage intake are key challenges for determining their potential for feeding in pasture based-dairy systems. We hypothesised that swards with greater WSC content would increase DMI and time spent grazing of dairy cows, thus improving pasture utilisation, increasing milk production and milk protein content, and reducing milk urea concentration due to an improved N use efficiency. The objective of this study was to preliminary examine the potential of pastures differing in their nutritional value, with emphasis on WSC and CP contents, to affect milk production and composition as well as the behaviour of dairy cows grazing PRG swards.

## 2. Materials and Methods

The study was carried out at the Campus “Pillanlelbún” (38°61′67″S, 72°46′67″W), La Araucanía region (Chile), owned by the Universidad Católica de Temuco, from September 2015 to May 2016. A one-hectare field was used for this pilot trial. 

### 2.1. Pasture Management and Treatments

In September 2015 (spring in the southern hemisphere), an area of 0.5 ha was sown with perennial ryegrass (*L. perenne* L.) cultivar Expo (cultivar bred for greater WSC contents, i.e., “high-sugar grass”) and 0.5 ha with the standard cultivar Extreme. Both are diploid cultivars and were sown at a rate of 25 kg seeds per hectare. 

In addition to the cultivars used, the two swards also varied in their fertilisation rate (partitioned in monthly applications from sowing onward) and the defoliation frequency. The high-sugar cultivar received an annual N fertilisation rate equivalent to 83.3 kg N/ha, and the defoliation occurred (either cutting for maintenance or grazing in the trial reported here) at the stage of three leaves per tiller, i.e., high-sugar (HS) treatment. On the other hand, the standard cultivar received an annual N fertilisation rate equivalent to 250 kg N/ha, and the defoliation occurred at the stage of two leaves per tiller, i.e., low-sugar (LS) treatment. 

From sowing to April 2016, swards were cut to a residual height of 5 cm (and the herbage harvested was removed from the plot) each time they reached the average number of leaves per tiller (2 or 3, respectively), and the fertiliser was partialized as reported by Rivero et al. [10]. In May 2016 (autumn in the southern hemisphere), both swards coincided in the date in which they achieved their average number of leaves per tiller, i.e., two for the LS treatment and three for the HS treatment. The grazing trial took place in this period.

### 2.2. Allocation and Management of Cows and Pasture

Twelve Holstein–Friesian cows with the following characteristics were used for the grazing trial: liveweight (LW) of 641 ± 119.2 kg, body condition score (BCS) of 3.5 ± 0.96 (scale from 1 to 5), days in milk (DIM) of 131 ± 35.3 days, number of lactations of 2.7 ± 1.56, and daily milk production of 21.2 ± 3.27 L/d on average (± SEM). Cows were split into two groups with similar average values of LW, BCS, DIM, number of lactations, and daily production, and each group was randomly allocated to one treatment (LS or HS). Before entering the experimental area, cows were grazing a PRG pasture in daily strips as part of a herd of 20 cows. 

Each 0.5 ha plot was divided into nine daily strips (~560 m^2^ each), and six cows grazed a daily strip (one LS and one HS) each day for 24 h (front and back electric fences were used for the daily allocation). The first three days were considered as a short acclimation period. The experimental period (observations and samplings) started on the fourth day, i.e., fourth daily strip. 

Cows were milked twice a day (morning and afternoon; no supplemental feed was provided), and milk production was recorded using a milk meter (Tru-Test Group, Auckland, New Zealand) in the milking parlour. On the last two milkings (afternoon milking on the last day and morning milking on the following day, i.e. just after the cows left the last strip), milk samples were collected (30 mL in each milking) from each cow (6 per treatment). Then, the two samples for each cow were pooled to produce a unique sample per cow. Samples were maintained in a cooler with icepack during their immediate transport to the lab for analysis. Water was always available in the grazing area.

### 2.3. Herbage Mass Assessment

Before cows entered the experimental strip (during morning milking), five samples were collected from each of the two strips to determine pre-grazing herbage mass (HM) using a 0.09 m^2^ frame (herbage was manually harvested to the ground level with scissors). The following morning, after the cows were removed from the grazed strip (for morning milking), five samples were collected from each of the two strips to determine post-grazing HM using the same procedure as for pre-grazing HM. This sampling was repeated for six days (samples from the six daily strips were collected in pre- and post-grazing). Herbage from these samples was weighed fresh, placed in paper bags, and oven-dried at 60 °C for 48–72 h until constant weight. Subsequently, the average value from the five samples collected per day and per strip was used to calculate the DM herbage mass per hectare (on pre- and post-grazing). Considering the area of each daily strip and the number of cows grazing, the herbage allowance (HA, kg DM available per cow), the average apparent daily DMI per cow, and the proportion harvested (in relation to the HA) were calculated for each of the six days. 

### 2.4. Forage and Milk Quality Evaluation

Snip samples were collected during the six days of the experimental period to determine nutritional composition. The day before each strip was grazed, in a one-hour window of time around noon, snip samples were manually cut at 5 cm above ground level following a W transect within the daily strip (9 points per strip). A pooled sample was produced for each treatment each day (~200 g fresh basis), placed in Ziploc bags, immediately frozen in liquid nitrogen, and kept in a cooler with icepack until immediate transport to the lab. Samples were kept frozen at −20 °C until freeze-drying and grinding (particle size: 2 mm) and analysed for WSC [17], CP (Kjeldahl method, N × 6.25) [18], neutral detergent fibre (NDF) [19], and acid detergent fibre (ADF) [20]. Representative subsamples of milk were analysed for fat, protein, lactose, and urea concentrations by infrared spectrophotometer (MilkoScan™ 4300; Foss Electric, Hillerod, Denmark). 

### 2.5. Behavioural Observations

Behaviour of cows grazing on each sward type was recorded every 10 min from 9:30 to 15:30 (between morning and afternoon milkings). For this purpose, cows were individually identified with a number (from 1 to 6 within each group) drawn with livestock markers in their flanks. One observer situated outside the grazing area recorded the activity each cow was performing at each observation time: grazing, ruminating, lying down, standing, or other (e.g., walking, drinking water, urinating, etc.). These observations were performed for the six experimental days (6 h a day).

### 2.6. Statistical Analysis

For analysing the variables related to the pasture (herbage mass, apparent DMI, proportion consumed, and forage nutritional composition) and the animal behaviour (percentage of time performing each activity between a.m. and p.m. milkings), a randomised complete block design was used, and a one-way analysis of variance (ANOVA) was performed. The daily strip (6 in total for the experimental period) was considered the block. For analysing milk production and milk composition, a completely randomised design was used, and a one-way ANOVA was performed. In this case, the cow was considered the replicate. For the behavioural pattern, an ethogram (one observation every 10 minutes) was produced. In this case, descriptive statistics was used to present the mean values across the six days for each observation time. All analyses were performed using Genstat 18 (©VSN International Ltd., Hemel Hempstead, UK).

## 3. Results

As intended with cultivar selection and pasture management, swards differed in their nutritional composition (Table 1). The HS sward had 44% more WSC (*p* = 0.002), 24% less CP (*p* < 0.001), almost double the WSC-to-CP ratio (*p* < 0.001), 17% less NDF (*p* = 0.024), and 9% less ADF (*p* = 0.008) than the LS sward.

Pre-grazing HM and HA were similar between swards, averaging 3806 kg DM/ha and 37.3 kg DM/cow, respectively, while post-grazing HM was 17% lower (*p* = 0.047) in the HS than in the LS sward (Table 2). However, the average apparent DMI estimated per cow did not vary between swards, and the overall average was 18.2 kg DM/d. The proportion of herbage removed tended (*p* = 0.068) to be greater in the HS sward (Table 2).

**Figure 1:**
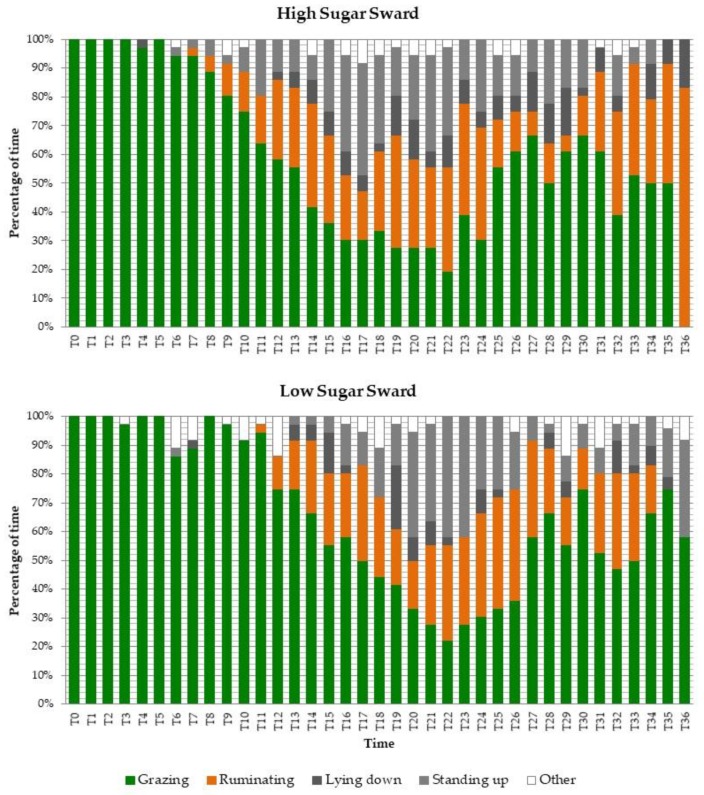
Ethogram of dairy cows grazing on a perennial ryegrass pasture with high (32.2% DM) and low (22.4% DM) contents of water-soluble carbohydrates. T0 corresponds to 9:30 and T36 corresponds to 15:30 (observations performed every 10 minutes by one observer). Bars are averages over six days.

The time spent grazing was greater (*p* = 0.003) and the time standing was lower (*p* = 0.008) in the LS sward than in the HS sward (Table 2). However, rumination time did not differ between swards, averaging 14.6% of their time between milkings. From the ethograms presented in Figure 1, it can be observed that cows in the HS sward spent the first hour in the new strip actively grazing, while rumination events started from the seventh observation when cows also started standing (not displaying other activity). However, in the LS sward, the cows actively grazed for longer, presenting the first rumination events at the 11th observation (almost two hours after cows started grazing the new strip), with lying down also being displayed in that period. Both groups presented a second peak of grazing events on the last two hours before the afternoon milking. 

Regarding milk production and quality, sward type did not affect any of the variables measured (Table 3). Taking the average of the two groups, milk production was 19.7 L/d per cow, and the milk contained 4.41% fat, 3.52% protein, 4.70% lactose, and 24 mg/dL urea.

## 4. Discussion

### 4.1. Nutritional Composition of Forage

The greater WSC content of the HS sward, achieved by less frequent defoliations and a lower N fertilisation rate compared with the LS sward, is supported by the WSC reserves, which are completely restored after the two-leaf stage [21,22]. The greater herbage WSC content observed in the HS sward (averaging 322 g/kg DM) compared with the LS sward agrees with the greater WSC content in AberMagic, a high-sugar cultivar, compared with the standard cultivar Arrow observed by Turner et al. [23] under cool temperate Tasmanian conditions. The differences shown in WSC content and WSC-to-CP ratio by the high-sugar trait in studies performed in Australia and New Zealand [24,25] has been inconsistent, compared with a more consistent effect shown in European studies [8,26]. This has previously been attributed to a genotype–environment interaction [12], with temperatures lower than 10 °C required for the expression of the trait [24]. A study performed in Chile evaluating HSG and standard PRG cultivars submitted to contrasting agronomic management systems (applying the same N fertilisation rates and defoliation frequencies as the present study) reported no consistent expression of the “high-sugar” trait [10]. Another study carried out in similar climate conditions as the current work did not show an expression of the high-sugar trait in diploid PRG cultivars selected for high WSC content defoliated at the stage of three leaves per tiller [27]. 

Regarding the average forage CP content, the HS sward showed a lower value compared with the LS sward, with a difference of 48 g/kg DM between swards, which was slightly lower than the difference reported by Rivero et al. [10] (56 g/kg DM) using the same high-sugar and standard diploid cultivars (developed in New Zealand) submitted to the same management system as the present study (191 vs. 247 g/kg DM for HS and LS, respectively) in autumn swards. When relating these two nutrients, the WSC-to-CP ratio in the HS sward surpassed 1.5, which is the value recommended by Parsons et al. [13] for a more efficient N partitioning under temperate conditions (New Zealand), although both cultivars were well above the critical 0.7–0.75 value, which is the value suggested by Edward et al. [12] to achieve a more efficient N partitioning. 

The greater WSC content in the HS sward found in the current study probably occurred at the expense of both CP and NDF content, as shown by the lower NDF and CP contents with increased WSC in the HS sward compared to the LS sward [6,28,29]. The difference in the NDF content in the current study between swards was 21 g/kg DM. This is consistent with the differences between high- and low-sugar cultivars reported by Smith et al. [30], which ranged from 30 to 50 g/kg DM. Nevertheless, the NDF content of the standard cultivar at the two-leaf stage was relatively high compared with the value reported by Rivero et al. [10] (390 g/kg DM) for LS swards (diploid and tetraploid standard cultivars) under the same agronomic management system in autumn. 

### 4.2. Herbage Masses and Apparent Dry Matter Intake

Pre-grazing HM was unaffected by sward type, but the dairy cows grazing the HS sward resulted in a greater grazing intensity, as evidenced by the lower post-grazing HM (408 kg DM ha^−1^ lower) and the trend towards a greater proportion of forage removed as a fraction of the forage offered. Cows consumed the HS sward faster than the LS sward, spending 4.7% less time grazing than in the LS sward. The lower fibre content observed in the HS sward could have led to a faster DM degradation and better digestibility, and this may have therefore produced either a greater biting rate or bite mass (or both) in the HS sward [31]. 

Herbage allowance is one of the most important sward characteristics that influence herbage DMI [32]. On average, daily HA for both swards in the present study exceeded 30 kg DM/cow. Thus, the herbage DMI was unaffected by sward type and was similar to the value reported by Bargo et al. [33], who recorded 20 kg DMI/cow with a daily HA of 40 kg DM/cow for non-supplemented mid-lactation dairy cows. Despite the difference observed in the nutritional value of the swards, it would not have been sufficient to impact herbage DMI, which could be related to an increased selection of green leaf by cows and more digestible herbage consumed with greater CP and lower NDF content compared to the forage on offer [34] as a way to increase the nutritional value of ingesta. In another short-term study (three weeks) performed under stall-feeding conditions, Moorby et al. [14] found that early-lactation dairy cows fed on HSG forage consumed 2.2 kg DM d^−1^ more herbage than those offered a standard PRG forage, with differences in WSC content (82 g kg^−1^ greater in the HSG) and NDF (83 g kg^−1^ lower in the HSG) between grass types. However, interpretation should be done with caution because the opportunity for diet selection in that study was limited by zero grazing, and cows were consequently not able to select more digestible diets with greater CP and lower NDF contents than the mean contents. 

### 4.3. Cows Behaviour

When herbage availability and accessibility is not a constraint, grazing behaviour is influenced by several factors related to the pasture, such as sward density [35] and sward surface height [36] as well as morphological [37] and chemical composition of the herbage offered [8], with the latter considered to have the largest impact on preference behaviour [38]. Furthermore, the energy status of dairy cows may alter grazing preference and ingestive behaviour [39]. Dairy cows’ preference is positively correlated with WSC content and negatively correlated with NDF content in perennial ryegrass cultivars [40]. Thus, an elevated content of WSC in grass with lower fibre content may increase forage digestibility [41], increasing herbage intake and availability of readily degradable energy [42]. 

Cows managed under strip-grazing regime ate the bulk of allocated herbage during the first hours of grazing, as evidenced by the greater grazing activity shown during the first two hours after the start of the grazing session in both sward types. However, the grazing time was reduced by 11 seconds for each g/kg DM of increase in WSC content. This could be explained by the findings of Shipley et al. [43], who stated that, if an animal must stop to select a bite, this increases its grazing time and the energy expended eating. 

### 4.4. Milk Production and Quality

Despite differences in nutrient content between the two swards, this study showed that milk production and quality were unaffected by increased forage WSC content in a high-quality autumn pasture. The absence of differences in milk production between high-sugar and low-sugar swards has been previously reported [1,14,26,28] and might be related to the fact that the apparent herbage DMI is unaffected by PRG sward type. Moreover, this lack of effect of WSC content on milk yield has been previously attributed to the fact that DMI is sensitive to the forage nutritional quality, which depends on whether the WSC content of grass is increased at the expense of CP, NDF, or both. Ellis et al. [44] analysed the effect of HSG on predicted milk yield and N excretion (including milk N) using a dynamic model and published literature data. Simulation results showed that the response of N use efficiency (milk N, in relation to N intake) and milk yield to increased WSC content was also driven by concurrent changes in the CP and NDF fractions. The greatest benefits in terms of N utilisation were found when forage WSC content was increased at the expense of CP content alone. In relation to milk production, the greatest increases were found when WSC increased at the expense of NDF, whereas it increased only slightly when WSC increased at the expense of both NDF and CP, and it decreased when WSC increased at the expense of CP content of grass (regardless of whether or not the grass had been fertilised with extra N). 

A greater WSC content in grass has been shown to optimise rumen microbial protein synthesis, improving the efficiency of ruminal NH_3_ and dietary N utilisation [4,5]. However, there was no evidence of any effect of high WSC content in grass on milk fat, milk protein, and milk urea content of dairy cows in mid-lactation in the present study. 

## 5. Conclusions

Selection of cultivars, along with management strategies, i.e., nitrogen fertilisation rates and defoliation frequencies, are effective tools to modify the nutritional composition of perennial ryegrass. Although the grazing behaviour was affected by the sward type, the differences in forage nutritional composition were not enough to impact dry matter intake, milk production, measured across six days, or milk components (including milk urea) in this short-term pilot trial. This could imply that high-quality pastures would require greater differences in nutritional composition to have a significant impact on animal performance.

## Figures and Tables

**Table 1:** Nutritional composition (in g kg^−1^ dry matter (DM), except for the WSC-to-CP ratio) of perennial ryegrass swards sown with either a high-sugar (HS) cultivar (Expo) grazed at three leaves per tiller and fertilised with an annual N fertilisation rate equivalent to 83.3 kg N/ha or a standard cultivar (Extreme) grazed at two leaves per tiller and fertilised with an annual N fertilisation rate equivalent to 250 kg N/ha (LS) in autumn.

Component	WSC	CP	WSC:CP	NDF	ADF
HS	322	154	2.1	379	206
LS	224	202	1.11	456	227
*p* Value	0.002	<0.001	<0.001	0.024	0.008

WSC: water-soluble carbohydrates; CP: crude protein; NDF: neutral detergent fibre: ADF: acid detergent fibre.

**Table 2:** Herbage mass (pre- and post-grazing), average herbage allowance, apparent dry matter (DM) intake, and behaviour of dairy cows grazing on a perennial ryegrass pasture with high (32.2% DM, HS) and low (22.4% DM, LS) contents of water-soluble carbohydrates.

Variable	HS	LS	SEM	*p* Value
*Pasture herbage mass and intake*				
DM herbage mass pre-grazing (kg/ha)	3694	3917	89.3	0.139
DM herbage mass post-grazing (kg/ha)	1735	2143	110.1	0.047
DM daily herbage allowance (kg DM/cow)	36.1	38.5	2.92	0.577
Average apparent DM intake per cow (kg DM/cow)	19.1	17.2	0.89	0.183
Proportion consumed	0.53	0.45	0.026	0.068
*Grazing behaviour (% time between milkings)*				
Grazing	66.9	71.6	1.07	0.003
Ruminating	15.4	13.7	0.99	0.216
Lying down	11	8.9	0.87	0.092
Standing	4.6	2.7	0.49	0.008
Other activities	2.2	3.2	0.36	0.067

**Table 3:** Milk production and composition from dairy cows grazing on a perennial ryegrass pasture with high (32.2% DM ^1^, HS) and low (22.4% DM, LS) contents of water-soluble carbohydrates ^1^.

Milk Component ^2^	HS	LS	SEM	*p* Value
Milk production (L/d)	19.3	20.0	2.60	0.848
Milk fat concentration	4.57	4.24	0.185	0.231
Milk protein concentration	3.52	3.52	0.126	0.985
Lactose concentration	4.71	4.68	0.106	0.862
Non-fat solids	9.28	9.24	0.479	0.874
Total solids	14.29	13.90	0.236	0.275
Milk urea (mg/dL)	21	26	2.5	0.258

^1^ DM: dry matter; ^2^ (% g/g) unless another unit stated

## References

[1] Balocchi O.A., López I.F. (2009). Herbage production, nutritive value and grazing preference of diploid and tetraploid perennial ryegrass cultivars (*Lolium perenne* L.). Chilean J. Agric. Res..

[2] Hills J.L., Wales W.J., Dunshea F.R., Garcia S.C., Roche J.R. (2015). Invited review: An evaluation of the likely effects of individualized feeding of concentrate supplements to pasture-based dairy cows. J. Dairy Sci..

[3] Nichols K., Dijkstra J., van Laar H., Pacheco S., van Valenberg H.J., Bannink A. (2019). Energy and nitrogen partitioning in dairy cows at low or high metabolizable protein levels is affected differently by postrumen glucogenic and lipogenic substrates. J. Dairy Sci..

[4] Hristov A.N., Jouany J.P., Pfeffer E., Hristov A.N. (2005). Factors affecting the efficiency of nitrogen utilization in the rumen. Nitrogen and Phosphorus Nutrition of Cattle.

[5] Lee M.R.F., Merry R.J., Davies D.R., Moorby J.M., Humphreys M.O., Theodorou M.K., MacRae J.C., Scollan N.D. (2003). Effect on increasing availability of water-soluble carbohydrates on in vitro rumen fermentation. Anim. Feed Sci. Technol..

[6] Miller L.A., Moorby J.M., Davies D.R., Humphreys M.O., Scollan N.D., MacRae J.C., Theodorou M.K. (2001). Increased concentration of water-soluble carbohydrate in perennial ryegrass (*Lolium perenne* L.): Milk production from late-lactation dairy cows. Grass For. Sci..

[7] Rasmussen S., Parsons A.J., Xue H., Newman J.A. (2009). High sugar grasses-harnessing the benefits of new cultivars through growth management. Pr. N. Z. Grassl. Assoc..

[8] Taweel H.Z., Tas B.M., Smit H.J., Elgersma A., Dijkstra J., Tamminga S. (2006). Grazing behaviour, intake, rumen function and milk production of dairy cows offered *Lolium perenne* containing different levels of water-soluble carbohydrates. Livest. Sci..

[9] Loaiza P.A., Balocchi O., Bertrand A. (2017). Carbohydrate and crude protein fractions in perennial ryegrass as affected by defoliation frequency and nitrogen application rate. Grass Forage Sci..

[10] Rivero M.J., Balocchi O.A., Moscoso C.J., Siebald J.A., Neumann F.L., Meyer D., Lee M.R.F. (2019). Does the “high sugar” trait of perennial ryegrass cultivars express under temperate climate conditions?. Grass Forage Sci..

[11] Humphreys M. (2006). Water-soluble carbohydrates in perennial ryegrass breeding. Grass Forage Sci..

[12] Edwards G.R., Parsons A.J., Rasmussen S., Bryant R.H. (2009). High sugar ryegrasses for livestock systems in New Zealand. Pr. N. Z. Grassl. Assoc..

[13] Parsons A.J., Edwards G.R., Newton P.C., Chapman D.F., Caradus J.R., Rasmussen S., Rowarth J.S. (2011). Past lessons and future prospects: Plant breeding for yield and persistence in cool-temperate pastures. Grass Forage Sci..

[14] Moorby J.M., Evans R.T., Scollan N.D., MacRae J.C., Theodorou M.K. (2006). Increased concentration of water-soluble carbohydrate in perennial ryegrass (*Lolium perenne* L.). Evaluation in dairy cows in early lactation. Grass Forage Sci..

[15] Taweel H.Z., Tas B.M., Smit H.J., Elgersma A., Dijkstra J., Tamminga S. (2005). Effects of feeding perennial ryegrass with an elevated concentration of water-soluble carbohydrates on intake, rumen function and performance of dairy cows. Anim. Feed Sci. Technol..

[16] Rivero M.J., Balocchi O.L., Neumann F.L., Siebald J.A. (2019). Grazing Preference of Dairy Cows and Pasture Productivity for Different Cultivars of Perennial Ryegrass under Contrasting Managements. Animals.

[17] MAFF (Ministry of Agriculture, Fisheries and Food) (1985). The Analysis of Agricultural Material.

[18] Bateman J.V. (1970). Nutrición Animal: Manual de Métodos Analíticos.

[19] Van Soest P.J., Robertson J.B., Lewis B.A. (1991). Methods for Dietary Fiber, Neutral Detergent Fiber, and Nonstarch Polysaccharides in Relation to Animal Nutrition. J. Dairy Sci..

[20] AOAC (1996). Official Methods of Analysis of AOAC International.

[21] Fulkerson W.J., Donaghy D.J. (2001). Plant-soluble carbohydrate reserves and senescence - key criteria for developing an effective grazing management system for ryegrass-based pastures: A review. Aust. J. Exp. Agr..

[22] Turner L.R., Donaghy D.J., Lane P.A., Rawnsley R.P. (2006). Effect of defoliation management, based on leaf stage, on perennial ryegrass (*Lolium perenne* L.), prairie grass (*Bromus willdenowii* Kunth.) and cocksfoot (*Dactylis glomerata* L.) under dryland conditions: 1. Regrowth, tillering and water-soluble carbohydrate concentration. Grass Forage Sci..

[23] Turner L.R., Donaghy D.J., Pembleton K.G., Rawnsley R.P. (2015). Longer defoliation interval ensures expression of the “high sugar” trait in perennial ryegrass cultivars in cool temperate Tasmania, Australia. J. Agric. Sci..

[24] Parsons A.J., Rasmussen S., Xue H., Newman J.A., Anderson C.B., Cosgrove G.P. (2004). Some ‘high sugar grasses’ don’t like it hot. Proc. N.Z. Grassl. Assoc..

[25] Cosgrove G.P., Burke J.L., Death A.F., Hickey M.J., Pacheco D., Fraser K., Lane G.A. (2007). Ryegrass with increased water soluble carbohydrate: Evaluating the potential for grazing dairy cows in New Zealand. Proc. N.Z. Grassl. Assoc..

[26] Tas B.M., Taweel H.Z., Smit H.J., Elgersma A., Dijkstra A., Tamminga T. (2006). Utilisation of N in perennial ryegrass cultivars by stall-fed lactating dairy cows. Livest. Sci..

[27] Moscoso C.J., Morgan S.A., Rivero M.J. (2019). The effect of drying methods on water-soluble carbohydrates and crude protein concentrations and their ratio in two perennial ryegrass cultivars. Agronomy.

[28] Tas B.M., Taweel H.Z., Smit H.J., Elgersma A., Dijkstra A., Tamminga T. (2005). Effects of perennial ryegrass cultivars on intake, digestibility and milk yield in dairy cows. J. Dairy Sci..

[29] Staerfl S.M., Amelchanka S.L., Kälber T., Soliva C.R., Kreuzer M., Zeitz J.O. (2012). Effect of feeding dried high-sugar ryegrass (‘AberMagic’) on methane and urinary nitrogen emissions of primiparous cows. Livest. Sci..

[30] Smith K.F., Simpson R.J., Oram R.N., Lowe K.F., Kelly K.B., Evans P.M., Humphreys M.O. (1998). Seasonal variation in the herbage yield and nutritive value of perennial ryegrass (*Lolium perenne* L.) cultivars with high or normal herbage water-soluble carbohydrate concentrations grown in three contrasting Australian dairy environments. Aust. J. Exp. Agr..

[31] Taweel H.Z. (2004). Perennial ryegrass for dairy cows: Grazing behaviour, intake, rumen function and performance. Ph.D. Thesis.

[32] Wilkinson J.M., Lee M.R.F., Rivero M.J., Chamberlain T. (2019). Some challenges and opportunities for grazing dairy cows on temperate pastures. Grass Forage Sci..

[33] Bargo F., Muller L.D., Delahoy J.E., Cassidy T.W. (2002). Milk response to concentrate supplementation of high producing dairy cows grazing at two pasture allowances. J. Dairy Sci..

[34] Dalley D.E., Roche J.R., Grainger C., Moate P.J. (1999). Dry matter intake, nutrient selection and milk production of dairy cows grazing rainfed perennial pastures at different herbage allowances in spring. Aust. J. Exp. Agr..

[35] Laca E.A., Ungar E.D., Seligman N., Demment M.W. (1992). Effects of sward height and bulk-density on bite dimensions of cattle grazing homogeneous swards. Grass Forage Sci..

[36] Griffiths W.M., Hodgson J., Arnold G.C. (2003). The influence of sward canopy structure on foraging decisions by grazing cattle. I. Patch selection. Grass Forage Sci..

[37] Dumont B., Petit M., D’hour P. (1995). Choice of sheep and cattle between vegetative and reproductive cocksfoot patches. Appl. Anim. Behav. Sci..

[38] Heady H.F. (1964). Palatability of herbage and animal preference. J. Range Manage..

[39] Gregorini P., Clark C.E.F., Jago J.G., Glassey C.B., McLeod K.L.M., Romera A.J. (2009). Restricting time at pasture: Effects on dairy cow herbage intake, foraging behavior, hunger-related hormones, and metabolite concentration during the first grazing session. J. Dairy Sci..

[40] Smit H.J., Tamminga S., Elgersma A. (2006). Dairy cattle grazing preference among six cultivars of perennial ryegrass. Agron. J..

[41] Van Soest P.J. (1994). Nutritional Ecology of the Ruminant.

[42] Cosgrove G.P., Edwards G.R., Rattray P.V., Brookes I.M., Nicol A.M. (2007). Control of grazing intake. Pastures and Supplements for Grazing Livestock.

[43] Shipley L.A., Spalinger D.E., Gross J.E., Hobbs N.T., Wunder B.A. (1996). The dynamics and scaling of foraging velocity and encounter rate in mammalian herbivores. Funct. Ecol..

[44] Ellis J.K., Dijkstra J., Bannik A., Parsons A.J., Rasmussen S., Edwards G.R., Kebreab E., France J. (2011). The effect of high-sugar grass on predicted nitrogen excretion and milk yield simulated using a dynamic model. J. Dairy Sci..

